# The role of IL-1B in breast cancer bone metastasis

**DOI:** 10.1016/j.jbo.2024.100608

**Published:** 2024-05-14

**Authors:** Jiabao Zhou, Penelope D. Ottewell

**Affiliations:** Division of Clinical Medicine, University of Sheffield, Beech Hill Road, Sheffield S10 2RX, United Kingdom

**Keywords:** Breast cancer, Bone metastasis, Interleukin-1B

## Abstract

Interleukin-1B (IL-1B) is a potent pro-inflammatory cytokine that plays multiple, pivotal roles, in the complex interplay between breast cancer cells and the bone microenvironment. IL-1B is involved in the growth of the primary tumours, regulation of inflammation within the tumour microenvironment, promotion of epithelial to mesenchymal transition (EMT), migration and invasion. Moreover, when breast cancer cells arrive in the bone microenvironment there is an upregulation of IL-1B which promotes the creation of a conducive niche for metastatic breast cancer cells as well as stimulating initiation of the vicious cycle of bone metastasis. Pre-clinical studies have demonstrated that inhibition of IL-1 signalling reduces bone metastasis from oestrogen receptor positive/triple-negative breast cancers in various mouse models. However, effects on primary tumours and soft tissue metastasis remain controversial with some studies showing increased tumour growth in these sites, whilst others show no effects. Notably, combining anti-IL-1 therapy with standard-of-care treatments, such as chemotherapy and immunotherapy, has been demonstrated to reduce the growth of primary tumours, bone metastasis, as well as metastatic outgrowth in other organs. This review focuses on the mechanisms by which IL-1B promotes breast cancer bone metastasis.

## Highlights

• Promoting EMT, vascular and immunological changes that drive tumour cells out of the primary site.
• Regulates breast cancer cell homing to the bone microenvironment.
• Promotes expansion of the bone metastatic niche and outgrowth of tumour cells disseminated in bone.
• Stimulates the vicious cycle of bone metastases.

## 1 Introduction

Bone is the most common metastatic site for advanced breast cancer, occurring in up to 70 % of those with metastatic disease. Breast cancer bone metastasis is currently incurable, with a median survival of 36.0 months and a 5-year survival rate of approximately 10 % [1]. Interleukin-1B (IL-1B) is a pro-inflammatory cytokine with well documented roles in regulating inflammation [2]. Over the last 10-years there have been an accumulation of data from clinical and pre-clinical studies demonstrating clear roles for this cytokine in promoting breast cancer metastasis and metastatic outgrowth of tumour cells in bone. Furthermore, there is a growing amount of evidence suggesting that inhibiting IL-1B production or activation of its’ receptor, IL-1R1, maybe effective therapeutic strategies for preventing/treating breast cancer bone metastasis [3], [4], [5], [6], [7], [8], [9]. In this review, we first discuss the molecular mechanisms by which IL-1B drives the metastatic cascade of breast cancer bone metastasis before exploring potential methods for targeting IL-1 signalling that may benefit patients in the future.

## 2 The IL-1B signalling pathway and its’ importance in breast carcinogenesis

**Fig. 1:**
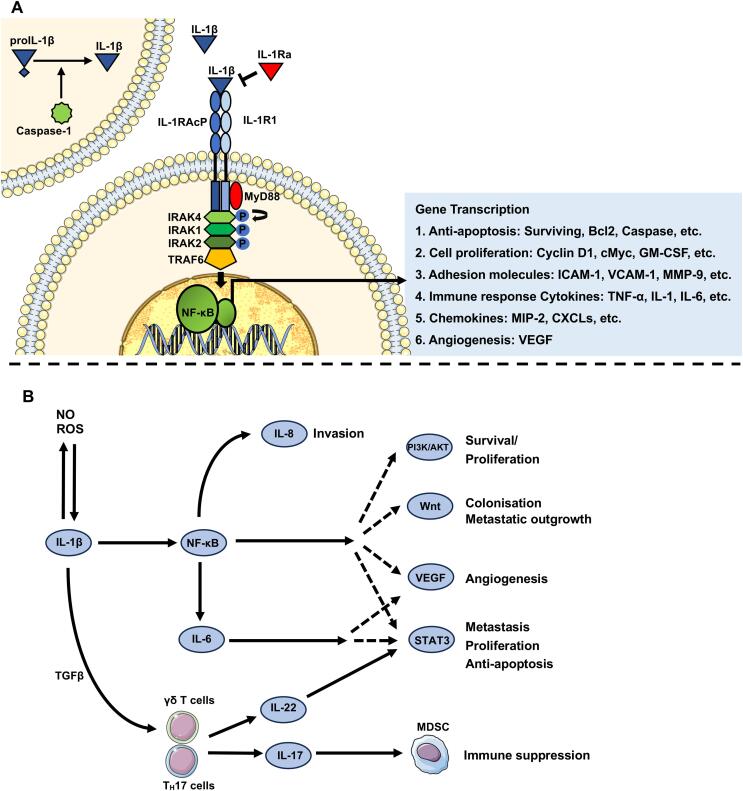
**A) IL-1B pathway: IL-1B is produced as inactive precursor pro-IL-1B in response to pro- inflammatory signals.** Activation of pro-IL-1B is initiated and cleaved by Caspase-1 to mature IL-1B. Mature IL-1B binds with IL-1R1 and IL-1RAcP to form a trimeric receptor complex. The trimeric complex rapidly recruits and assembles myeloid differentiation primary response gene 88 (MYD88) and interleukin-1 receptor-activated protein kinase 4 (IRAK4). Simultaneously, IRAK4 undergoes auto-phosphorylation, followed by the phosphorylation of IRAK1 and IRAK2 as well as recruitment and oligomerization of tumour necrosis factor–associated factor 6 (TRAF6). Finally, IRAK1/2 and TRAF6 dissociate from the receptor complex and transmit signals through adaptors and protein kinases to activate downstream nuclear factor-κB (NF-κB) signalling pathways. B) IL-1B and its downstream interleukins in mammary carcinogenesis: IL-1B facilitates the production of carcinogenic mediators, such as nitric oxide (NO) and reactive oxygen species (ROS). IL-1B directly induce proliferation, survival, stemness, epithelial–mesenchymal transition (EMT), migration and invasion in breast cancer by mediating excessive activation of NF-κB-, STAT3-, Wnt-, PI3K/AKT- signalling and induce angiogenesis by mediating VEGF. IL-1B increases breast cancer cell aggressiveness/invasiveness by upregulating IL-6/STAT3 and IL-8. IL-1B synergism with TGFβ induces differentiation of TH17 cells and γδ T cells, which triggers the secretion of pro-tumourigenic IL-17 and IL-22, which establish immunodepression microenvironment and exacerbate tumour development via activation of STAT3 signalling, respectively.

Chronic inflammation is one of key drivers for breast carcinogenesis, and IL-1 has long been associated with inflammation-driven carcinogenesis. The IL-1 (Interleukin-1) family is a 11-member group of cytokines or signalling proteins, with IL-1A and IL-1B being the most well-known: IL-1A is synthesized as a biologically active cytokine which is mainly localised within the nucleus and can be released during cell damage or necrosis. Whereas IL-1B is synthesized as an inactive precursor and requires cleavage by the enzyme Caspase-1 to become biologically active and is primarily located in the cytoplasm. IL-1B activation and release occur in response infection or inflammatory triggers and this molecule further triggers an intricate cascade of signalling molecules, including immune response cytokines, chemokines and adhesion molecules (Fig. 1A). This cascade is pivotal role to initiate and coordinate inflammatory responses, including regulating both innate and adaptive immunity, as well as regulating both anti- and pro- tumour effects.

In the context of chronic inflammation, IL-1B facilitates the production of carcinogenic mediators, such as nitric oxide (NO) and reactive oxygen species (ROS). Meanwhile, unregulated IL-1B directly initiates the downstream release of proinflammatory cytokines, including IL-6, IL-8, IL-17, exacerbating inflammation-induced carcinogenesis (Fig. 1B) [10]. Whereas in carcinogenesis, IL-1B directly activates of nuclear factor-κB (NF-κB), and it can further enhance the activation of signal transducer and activator of transcription 3 (STAT3) signalling, Wnt signalling and PI3K/AKT signalling to induce tumour proliferation, survival, stemness, EMT/metastasis in addition to promoting angiogenesis via increasing vascular endothelial growth factor (VEGF) [8], [9], [10], [11], [12]. Furthermore, downstream induction of proinflammatory cytokines through increased IL-1B/NF-κB signalling can further enhance the aggressiveness of carcinomas. For example, upregulated IL-6 results in excessive activation of STAT3 inducing proliferation, anti-apoptosis, metastasis [12]; IL-8 induced by IL-1B can increases invasiveness and the metastatic potential of both ER- and ER+ breast cancers [13]. Moreover, IL-1B, in conjunction with TGFβ, induces the differentiation of T helper 17 (TH17) cells and γδ T cells, which triggers the secretion of pro-tumourigenic IL-17 and IL-22, where IL-17 promotes further MDSC recruitment leading to immunosuppression and IL-22 activates of STAT3 signalling [10], [14]. Taken together, it appears that activation of proinflammatory cytokines all have the potential to promote carcinogenesis, but IL-1B seems to be the dominant cytokine that drives these pro-tumour processes.

## 3 IL-1B in the metastatic cascade of breast cancer to bone and underlying mechanisms

**Fig. 2:**
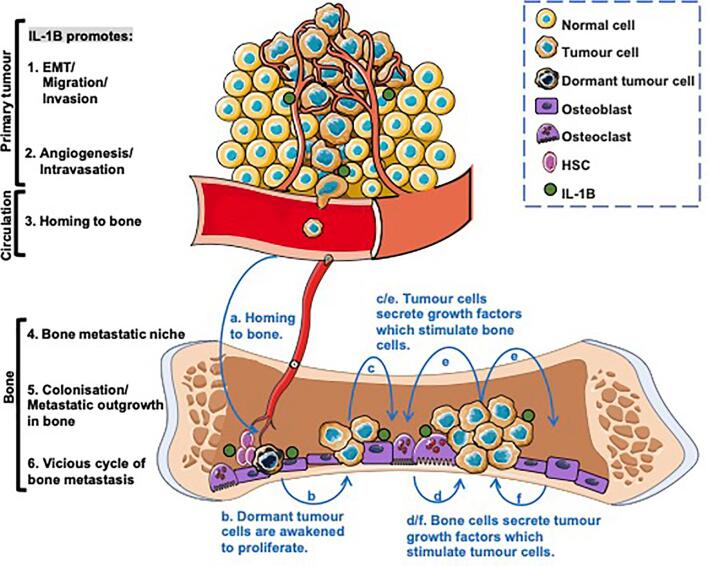
**The effects of IL-1B on breast cancer bone metastatic cascade and “vicious cycle of bone metastasis”** IL-1B plays multiple roles to promote breast cancer metastasise to bone: 1. In the primary site, IL-1B promotes EMT, migration and invasion. 2. IL-1B promotes angiogenesis and intravasation of tumour cells into the bloodstream. 3. IL-1B induces tumour bone homing. 4. IL-1B stimulates the activity of cells that make up the bone metastatic niche (osteoblast and perivascular endothelial cells) causing expansion of the niche. 5. IL-1B activates wnt signalling in cancer stem cells causing proliferation and metastatic outgrowth. 6. IL-1B stimulates osteoclastic bone resorption and promotes the vicious cycle of bone metastasis where blue a-f indicate breast cancer induced osteolytic “vicious cycle of bone metastasis”. (For interpretation of the references to colour in this figure legend, the reader is referred to the web version of this article.)

Bone metastasis is a complex, multistage process [15]: Initially, cancer cells from the primary tumour trigger epithelial-to-mesenchymal transition (EMT), allowing them to migrate, invade the surrounding tissue and enter the circulatory system. Once in the circulation, cancer cells disseminate to bone where they exit from blood vessels and settle in the distant metastatic niche. Subsequently, these tumour cells enter a dormant state or proliferate to form overt metastatic tumours. IL-1B has been shown to play important role in all of these processes (Fig. 2).

### 3.1 IL-1B facilities EMT, migration, invasion and angiogenesis

Tumour cells undergo epithelial–mesenchymal transition (EMT) and then acquire migratory and invasive ability is the first step of metastatic cascade. Tulotta *et al.,* found that genetic overexpression of IL-1B in ER + ve (T47D-IL-1B+ and MCF7-IL-1B+) and ER-ve (MDA-MB-231-IL-1B+) breast cancer cells led to both genotypic and phenotypic alterations associated with EMT (reduced E-cadherin, reduced γ-catenin and increased N-cadherin), compared with corresponding wildtype cells [4]. Furthermore, scratch assay and matrigel-coated/uncoated transwell assay showed that these IL-1B-overexpressing human breast cancer cell lines and IL-1B-overexpressing mouse triple negative breast cancer cells (E0771-IL-1B+) exhibited enhanced migratory and invasive capabilities, compared with corresponding wildtype cells [4], [5], [7]. Moreover, tumour derived IL-1B also triggered the paracrine feed-forward signalling to stimulate malignant invasive ability of cancer-associated fibroblasts (CAFs) [16]. In addition, adding exogenous IL-1B also facilitated infiltration and invasion of ER + ve, ER + ve, HER2 + ve human BT-474 breast cancer cells and increased stem-cell-like phenotypes, invasion of ER + ve MCF7 cells [12], [17]. These pro-metastatic effects of IL-1B were confirmed in experiments where inhibition IL-1B or IL-6 with an anti-IL-1B antibody or IL-1R antagonist Anakinra attenuated EMT, migratory and invasive capabilities of the aforementioned tumour cells [4], [12]. Interestingly, Zhou *et al.,* found that tumour derived IL-1B increased the ability of mouse E0771 and Py8119 cells to migrate and invade to human pre-osteoblast OB1 cells, but exerted reduced migratory/invasive effects towards human umbilical vein endothelial cells (HUVEC) [7]. These data suggest that the pro-metastatic effects of IL-1B may be organ-specific.

During the processes of extravasation, intravasation and metastatic outgrowth from the vascular niche, tumour cells must interact with the vasculature. IL-1B has been shown to facilitate tumour angiogenesis to promote tumour growth and distant metastasis. *In vitro* and *in vivo* studies showed that IL-1B upregulated the expression of angiogenesis biomarkers, such as VE-cadherin, VEGFR-1, by p38/MAPK and PI3K/Akt signalling pathways and it has synergetic effects with VEGF to facilitate early angiogenesis [18], [19].

In agreement with the data presented above breast cancer patients whose primary tumours express IL-1B have increased likelihood of developing subsequent metastases [3], [4]. For these patients early (neo-adjuvant) intervention with an IL-1 targeted therapy, such as Anakinra, may prevent escape of tumour cells from the primary site reducing the probability of future metastases. This hypothesis warrants further investigation.

### 3.2 IL-1B facilitates breast cancer cell extravasation and homing to the bone marrow

The process of tumour cell extravasation and homing to the bone marrow is a critical step in the formation of bone metastases. Once tumour cells circulate in the bloodstream, they first need to adhere to the endothelial cells lining the blood vessels within the bone marrow. After adhesion, cancer cells undergo extravasation to migrate through the endothelial cell layer and enter the bone marrow. Subsequently, these tumour cells are guided by chemotactic signals toward the bone metastatic niche which comprises of the endosteal niche (comprising osteoblasts and haematopoietic stem cells) and the *peri*-vascular niche. Here the bone marrow microenvironment provides a supportive niche for cancer cell survival, proliferation or dormancy [20]. IL-1B from osteoblasts has been shown to facilitate adhesion of circulating tumour cells to sinusoidal endothelial cells by increasing the expression of vascular cell adhesion molecules such as intercellular adhesion molecule 1 (ICAM-1), vascular cell adhesion molecule 1 (VCAM-1), and E-selectin [21]. The CXCR-4/CXCL-12 axis plays a significant role in breast cancer bone metastasis, where CXCR-4 activation on the surface of breast cancer cells facilitates their adhesion to bone marrow endothelial cells, and it guides them to the bone due to the attraction of CXCL-12 produced by bone marrow stromal cells and osteoblasts. Valdivia-Silva *et al.,* found that IL-1B increased the expression of CXCR-4 of MCF-7 cells that, in turn, had a higher migration index to CXCL-12 [22]. In addition, IL-1B from MDA-MB-231 cells activated mesenchymal stem cells, leading to the induction of CXCL-12, which, in turn, promoted the *trans*-endothelial migration of these breast cancer cells into the bone [23]. To identify and distinguish between molecular expression profiles that are specific to breast cancer homing to bone and bone colonisation, Nutter *et al.,* found that MDA-IV cells, that have acquired a higher ability to home to bone through repeated *in vivo* passaging, had higher levels of *IL1B* gene expression and IL-1B secretion, compared with parental MDA-P (parental) cells [4]. These data strongly suggest that IL-1B is correlated with breast cancer homing to bone. Moreover, IL-1B has been confirmed a strong stimulator of bone resorption *in vitro* and *in vivo* [24]. IL-1B can upregulate the production of RANKL enhancing its activity and stimulating osteoclastogenesis. Meanwhile, this cytokine stimulates an inflammatory cascade: IL-1B stimulates T cells and macrophages in both autocrine and paracrine fashions amplifying the inflammatory response, in turn these activated immune cells produce more IL-1B and RANKL, which induce macrophage transdifferentiate toward preosteoclasts leading to the increased bone resorption [24]. Tulotta *et al.,* found that tumour derived IL-1B can stimulate the expansion of the metastatic niche, increasing proliferation of blood vessels, osteoblasts, and haemopoietic stem cells thereby promoting tumour cell extravasation and metastatic outgrowth of tumour cells that disseminated in this site [4]. These data suggest that IL-1B may play an important role in inducing the formation of pre-metastatic niche that is beneficial to breast cancer bone metastasis. As advocated in section 3.1, data strongly indicate that early intervention with an IL-1 targeted treatment is likely to be of benefit to breast cancer patients. In this instance inhibiting IL-1 may prevent the formation of a pre-metastatic niche, rendering the bone microenvironment inhospitable for tumour cells preventing seeding of this site.

### 3.3 IL-1B facilitates dormant cell reactivation and vicious cycle of breast cancer bone metastasis

Once breast cancer cells enter bone, most of them do not directly proliferate and form metastatic lesions, instead enter a state of dormancy as a mechanism to help them survive until bone microenvironment is sufficiently permissive for proliferation and formation of metastatic lesions [15]. Clinical evidence shows disseminated tumour cells (DTCs) in the bone can be found before clinical detection of breast cancer at primary site and, importantly, around 30 % of breast cancer patients have DTCs in their bone marrow, and for most patients these cells will remain dormant for several months to years prior to proliferating into metastatic lesions [25]. Increased evidence indicates that bone-derived transforming growth factor-beta (TGF-β1) and IL-1B can reactivate dormant disseminated tumour cells (DTCs) stimulating metastatic outgrowth in the bone [26]. Sosnoski *et al.,* found that IL-1B and TNF-α significantly increased the growth/proliferation of previously dormant (MDA-MB-231BRMS1), MDA-MB-231 cells when co-cultured 3D with osteoblast *in vitro* [27].

Other *in vivo* evidence showed that administration of Anakinra for 3-days before or 7 days after intra-venous injection of DiD-labelled IL-1B overexpressing (MDA-MB-231-IV) cells had no effect on the number of DiD-labelled cells detected in bone, but inhibited proliferation of these cells in the bone, which suggests inhibition of IL-1B may limit tumour cell reactivation [8]. Additionally, a recent study indicated that bone marrow-derived IL-1B promoted breast cancer cell colonisation in the bone by activation of intracellular NF-κB/CREB signalling in breast cancer cells, resulting in autocrine Wnt signalling and cancer stem cell (CSC) colony formation [9]. These data suggest that IL-1B plays an important role in dormant cell reactivation and breast cancer metastatic colonisation in the bone. If we can find a way to permanently supress IL-1 secretion in the bone metastatic niche, this may provide a way to inhibit metastatic outgrowth in bone preventing future recurrence at this site.

### 3.4 IL-1B facilitates the ‘Vicious Cycle’ of breast cancer bone metastasis

In osteolytic lesions, there is a self-reinforcing and destructive process involving bone resorption and tumour growth, known as the “vicious cycle” [15], [20]. Awakened, proliferating DTCs release factors, such as parathyroid hormone-related protein (PTHrP), IL-1B and IL-6, IL-8 and IL-11 that stimulate osteoclast and osteoblast activity. Data suggest that IL-1B promotes osteoclastic bone resorption though activating osteoclasts, promoting osteoclast differentiation, multinucleation and survival [28]. IL-1B has been shown to drive osteoclast differentiation via a receptor activator of NF-κB ligand (RANKL)/RANK independent mechanism, with high concentrations of IL-1B in bone leading to a synergistic effect on RANKL induced osteoclast formation generating excessive bone destruction [28]. This excessive bone resorption, resulting in the release of calcium and growth factors, such as transforming growth factor-β (TGF-β) and insulin-like growth factors (IGFs), from the bone matrix. These released growth factors promote the proliferation of cancer cells in the bone, further fuelling tumour growth which, in turn, produce more PTHrP, IL-1B and IL-6 to consequently cause a positive feedback loop. Both *in vitro* and *in vivo* studies have demonstrated the key role of IL-1B in this ‘vicious cycle’: *In vitro* co-culture of breast cancer cells (MDA-MB-231 or T47D) with bone cells (human bone marrow stromal cell HS5 or pre-osteoblast cell OB1) not only significantly increase the proliferation of these cells, but also significantly increased *IL-1B* expression and secretion form all of these cell types [4]. Moreover, Loftus *et al.,* found that MDA-MB-231 or MCF7–derived extracellular vesicles (EVs) enhanced *IL-1B* expression and IL-1B secretion by osteoblasts *in vitro* [29]. Mice that were intra-cardiac or intra-venous injected with IL-1B-overexpressing MDA-MB-231 cells had significantly larger osteolytic lesions and a greater metastatic burden in bone, compared with mice injected with MDA-MB-231 wildtype cells [4]. In agreement, depletion of IL-1B from the microenvironment (using IL-1B^−/−^ C57BL/6 mice) prevented the development of osteolytic lesions or bone metastasis formation after intra-cardiac injection of E0771, compared with IL-1B^fl/fl^ control mice which developed large osteolytic metastasis [5]. Moreover, various breast cancer mouse models have consistently indicated that pharmacological inhibition of IL-1 signalling, by Anakinra or Canakinumab also reduced bone turnover resulting in higher % bone volume/tissue volume (BV/TV) in mouse tibias and reduced activity of both osteoclasts and osteoblasts compared with mice in control groups [4], [5], [7], [8], [9]. It therefore appears that IL-1B plays key roles in the vicious cycle of bone metastasis and inhibiting this molecule can prevent metastatic outgrowth of tumour cells disseminated in bone, however, the relative importance of this cytokine in the vicious cycle of bone metastasis remains to be determined.

### 3.5 The vicious cycle and beyond: Targeting IL-1B for the treatment of breast cancer bone metastases

Until recently it was widely believed that breaking the vicious cycle would be key to preventing/treating breast cancer bone metastasis. Clinically, the most effective way at inhibiting osteoclastic bone resorption is to give a RANK-L targeted antibody such as denosumab. In clinical trials denosumab has been shown to be highly effective at reducing osteoclast activity and tumour induced osteoclastic bone resorption in breast cancer patients with bone metastasis but has not shown any anti-tumour efficacy [30]. Furthermore, other anti-resorptive agents such bisphosphonates have no effects on overt metastases but can prevent future occurrence of bone metastasis [31], [32]. These data suggest that once tumours are established in bone inhibiting the vicious cycle through targeting osteoclasts alone is not sufficient to reduce tumour burden. However, a two-pronged attack of reducing bone turnover (the vicious cycle) and promoting anti-tumour immune responses may be the key to inhibiting metastatic outgrowth in bone or even reducing overt metastases. Administration of bisphosphonates results in apoptosis of osteoclasts, reduced osteoclast activity and an acute immune response in humans and mouse models [33]; addition of these drugs to standard of care treatments inhibits subsequent occurrence of bone metastasis in breast cancer patients [31], [32]. Administration of anti-IL-1 treatments, in mouse models, have similar effects reducing osteoclastic bone resorption and recruiting anti-tumour immune cells into the bone environment resulting in metastatic outgrowth being almost eliminated in bone [4], [5], [7], [8], [9]. However, it should be noted that experimentsutilising anti-IL-1 treatments have not yet been tested in humans and when given alone, anti-IL-1 treatments do not reduce the burden of existing bone metastasis and can increase growth of soft tissue tumours. Early data suggest that further increasing anti-tumour immune response with chemotherapy or immunotherapy drugs increase the anti-tumour effects of IL-1 targeted therapies further inhibiting bone metastasis and reducing soft tissue tumour burden, however, these are yet to be validated [5], [34]. Additional data are required to determine if effective combination therapies can be established to reduce overt bone metastases without adversely affecting soft tissue tumours before this strategy can be taken forward into clinical trials.

## 4 Conclusions and unanswered questions

Targeting IL-1B signalling may be an effective way to prevent occurrence of breast cancer metastasis. However, more effective combination treatments need to be established before IL-1B targeting can be used for the treatment of established bone metastases.

There are some key points that need to be addressed before we can consider targeting IL-1B as a novel treatment for breast cancer bone metastasis:

• Is targeting IL-1B after development of established bone metastases too late to achieve therapeutic benefit?
• Can IL-1B be targeted to prevent bone metastasis without adversely affecting tumour development in soft tissues?
• Does IL-1B need to be consistently suppressed to achieve therapeutic benefit; what happens to dormant cells after removal of anti-IL-1 therapy?

## CRediT authorship contribution statement

**Jiabao Zhou:** Writing – original draft. **Penelope D. Ottewell:** Writing – review & editing.

## Declaration of competing interest

The authors declare that they have no known competing financial interests or personal relationships that could have appeared to influence the work reported in this paper.
